# Recyclable SERS-Based Immunoassay Guided by Photocatalytic Performance of Fe_3_O_4_@TiO_2_@Au Nanocomposites

**DOI:** 10.3390/bios10030025

**Published:** 2020-03-16

**Authors:** Yuanyuan Du, Hongmei Liu, Yiran Tian, Chenjie Gu, Ziqi Zhao, Shuwen Zeng, Tao Jiang

**Affiliations:** 1 Department of Microelectronic Science and Engineering, School of Physical Science and Technology, Ningbo University, Ningbo 315211, China; 18895626819@163.com (Y.D.); 18232259365@163.com (Y.T.); guchenjie@nbu.edu.cn (C.G.); zhaoziqi@nbu.edu.cn (Z.Z.); 2 Institute of Solid State Physics, Shanxi Datong University, Datong 037009, China; lhm9898@163.com; 3 XLIM Research Institute, UMR 7252 CNRS/University of Limoges, Avenue Albert Thomas, 87060 Limoges, France; zeng@xlim.fr

**Keywords:** recyclable immunoassay, PSA, Fe_3_O_4_@TiO_2_@Au, Ag-coated sandpaper

## Abstract

A novel recyclable surface-enhanced Raman scattering (SERS)-based immunoassay was demonstrated and exhibited extremely high sensitivity toward prostate specific antigen (PSA). The immunoassay, which possessed a sandwich structure, was constructed of multifunctional Fe_3_O_4_@TiO_2_@Au nanocomposites as immune probe and Ag-coated sandpaper as immune substrate. First, by adjusting the density of outside Au seeds on Fe_3_O_4_@TiO_2_ core-shell nanoparticles (NPs), the structure-dependent SERS and photocatalytic performance of the samples was explored by monitoring and degradating 4-mercaptobenzonic acid (4MBA). Afterwards, the SERS enhancement capability of Ag-coated sandpaper with different meshes was investigated, and a limit of detection (LOD), as low as 0.014 mM, was achieved by utilizing the substrate. Subsequently, the recyclable feasibility of PSA detection was approved by zeta potential measurement, absorption spectra, and SEM images and, particularly, more than 80% of SERS intensity still existed after even six cycles of immunoassay. The ultralow LOD of the recyclable immunoassay was finally calculated to be 1.871 pg/mL. Therefore, the recyclable SERS-based immunoassay exhibits good application prospects for diagnosis of cancer in clinical measurements.

## 1. Introduction

Prostate cancer has become the second most common non-skin malignancies in men worldwide [1,2]. Therefore, to impede this pressing trend, it is essential to realize the early diagnosis and therapeutics of prostate cancer for which prostate specific antigen (PSA) has been the most extensively applied directional tumor marker [3]. Surface-enhanced Raman scattering (SERS) technique has attracted wide interest as a method extensively utilized in the field of labeled immunoassay for monitoring specific cancer antigens, mainly due to a number of advantages such as good spectral selectivity, no self-quenching and photobleaching, higher sensitivity, and multicomponent detection ability [4,5,6,7,8,9,10]. Accordingly, the ultrasensitive SERS-based immunoassays, which are formed of immune probe, target antigen, and antibody-immobilized substrate, have been built on the basis of representative sandwich immune structures [11,12,13]. It is known that noble metal nanomaterials with electromagnetic enhancement effect that primarily depends on their structures, sizes, and morphologies, are usually utilized in the immune probe and substrate for achieving ultrahigh sensitivity. Therefore, owing to their high cost for immune measurements and potential impact on the environment, it is promising to realize recycling of immune structures in clinical detection.

An effective solution that recycles detection of sandwich structures and saves resources is the introduction of nanomaterials with photocatalytic activity into the immune structures. Among them, semiconductor materials such as titanium dioxide (TiO_2_) with self-cleaning effect has been widely used for the degradation of microorganisms [14,15], cell debris [16], and antigens [17], in biological and water environments, owing to their nontoxicity, low price, and long-term chemical stability. However, the wide band gap of TiO_2_, i.e., 3.2 eV, together with the high recombination efficiency of electron-hole pairs, extremely limits the photocatalytic activity. On this basis, a series of strategies, for example, semiconductor coupling [18], ions doping [19,20], and deposition of noble metals [21,22,23] have been developed to improve their photocatalytic effect, which mostly depends on the degree, properties, and work function of the metal. Among these methods, the linked noble metal (Ag or Au) has a strong ability to absorb photogenerated electrons with different work functions, which inhibit the recombination of electron-hole pairs, and therefore endow TiO_2_ enhanced photocatalytic efficiency. For example, the photocatalytic performance of some Ag/TiO_2_ and Au/TiO_2_ hybrid materials for methanol and ethylene has been improved by nearly 100 times as compared with bare TiO_2_, which is attributed to the effective interfacial charge transfer from TiO_2_ to metal nanoparticles (NPs) under UV excitation, as demonstrated by in situ LSPR spectroscopy evaluation [24]. In addition, magnetic composition such as Fe_3_O_4_, which is famous due to its superparamagnetic and biocompatibility in the biomedical field, has also been introduced into photocatalytic materials for better separation of the recovered samples [25,26,27]. In spite of the fact that Fe_3_O_4_@TiO_2_@Au nanocomposites have been investigated in previous studies, few researches have focused on their clinical application in recyclable sandwich immunoassays.

Herein, a novel recyclable SERS-based immunoassay constructed of multifunctional Fe_3_O_4_@TiO_2_@Au nanocomposites as immune probe and Ag-coated sandpaper as immune substrate was applied to detect tumor marker PSA. First, the Fe_3_O_4_@TiO_2_@Au nanocomposites were synthesized by hydrothermal method and electrostatic adsorption, and the density of the outside Au seeds on the Fe_3_O_4_@TiO_2_ core-shell NPs was systematically investigated. The sample prepared by employing 100 mL synthetic stock solution of Au seeds was found to show the optimum SERS and photocatalytic performances, owing to the synergistic effect of the locked-in contact between Au seeds and Fe_3_O_4_@TiO_2_ NPs and effective electron transfer. Then, the SERS enhancement capability of Ag-coated sandpaper was evaluated by monitoring 4MBA and a limit of detection (LOD) of 0.014 mM was achieved. Afterwards, the feasibility of recyclable SERS-based immunoassay for tumor biomarker was demonstrated by monitoring 10^−7^ g/mL PSA antigen. More than 80% of the SERS intensity of the sandwich immune structure could still be maintained after six cycles of immunoassay. Consequently, the sandwich immune structure with only one batch of the noble metal nanostructures was applied to analyze a series of antigens with different concentrations and an ultralow LOD of 1.871 pg/mL was achieved. It is envisioned that this recyclable strategy which gets rid of the weaknesses of using numerous noble metal nanomaterials, has promising prospects in applications of clinical measurements for the diagnosis of cancer.

## 2. Materials and Methods

### 2.1. Chemicals

All the chemicals used in the preparation of the immune probe and substrate were illustrated as following: FeCl_3_·6H_2_O, ethylene glycol (EG), ethanol, aqueous ammonia (NH_3_∙H_2_O), acetonitrile, titanium oxide (TBOT), ammonium acetate (NH_4_Ac), 3-aminopropyltrimethoxysilane (APTMS), ammonium fluoride (NH_4_F), sodium citrate, phosphate buffer saline (PBS, pH = 7.0), 4-mercaptobenzonic acid (4MBA, 90%), hydrogen tetrachloroaurate (III) hydrate (HAuCl_4_· x H_2_O, 99.9%), Tris-buffered saline (TBS, pH = 7.6), TBS/0.05% Tween 20 buffer solution (pH = 8), bovine serum albumin (BSA), PSA (detection), anti-PSA (capture), alpha fetoprotein (AFP), and CA19-9. The chemicals used were analytical grade and Milli-Q water (18.2 MΩ·cm) was applied to prepare all synthesis solutions.

### 2.2. Preparation of the Fe_3_O_4_@TiO_2_@Au Immune Probes

#### 2.2.1. Synthesis of Fe_3_O_4_ NPs

In a typical hydrothermal method [28], 1.35 g of FeCl_3_·6H_2_O, 3.85 g of NH_4_Ac, and 0.4 g of sodium citrate were mixed in 70 mL of EG under stirring at 170 °C for 1 h. Then, the obtained solution was hydrothermally heated at 200 °C for 16 h. Finally, the natural cooled samples were separated and washed with ethanol and dried before use.

#### 2.2.2. Synthesis of Fe_3_O_4_@TiO_2_ NPs

The fabrication of Fe_3_O_4_@TiO_2_ nanoparticles was carried out according to a modified sol-gel strategy [29]. Briefly, as-prepared Fe_3_O_4_ was dissolved in 120 mL of acetonitrile/ethanol (volume ratio of 1:3), followed by adding the 375 µL of NH_3_∙H_2_O and 1 mL of TBOT under continuous stirring for 1.5 h. The resultant products were magnetically collected and thoroughly cleaned, followed by a hydrothermal reaction assisted by NH_4_F to 180 °C for 24 h to improve the crystallinity of the samples. After, the as-prepared product was magnetically separated and washed with deionized water for several times, it was finally stored in ethanol before use.

#### 2.2.3. Fabrication of Fe_3_O_4_@TiO_2_@Au Nanocomposites

A typical electrostatic adsorption technology was used for the preparation of Fe_3_O_4_@TiO_2_@Au nanocomposites [27]. First, the nanocomposites were amino modified by treating them with APTMS at 85 °C for 4 h. Meanwhile, the Au seeds were prepared through the reduction of metal ions by using sodium citrate in accordance with previous work [30]. Afterward, 200 µL ethanol solution of the amino treated samples were dropped into a 20 mL aqueous solution of Au seeds and mechanically stirred for 15 min. The resulted samples were separated by magnetism and washed with deionized water for 3 times. In addition, the surface density of the Au seeds was adjusted by changing the amount of Au seed solution (40, 60, 100, and 200 mL).

#### 2.2.4. Preparation of the Immune Probes

First, 20 µL of 4MBA (1 mM) was dropped into the solution of the as-obtained Fe_3_O_4_@TiO_2_@Au (synthesized with 100 mL of Au seed solution) before being incubated at 30 °C for 12 h, and the unattached 4MBA were cleaned by magnetic separation. Secondly, anti-PSA was coupled directly onto the 4MBA-labled Fe_3_O_4_@TiO_2_@Au nanocomposites via static and hydrophobic interactions [31,32]. The immobilization procedure was actualized for 3.5 h. Thereafter, the excess antibodies were removed by magnetic separation. In order to avoid nonspecific adsorption of antigen, the bare points on the 4MBA-labled Fe_3_O_4_@TiO_2_@Au nanocomposites were treated with a blocking solution (3 wt% BSA in PBS) at 30 °C for 1 h. The excess BSA was removed by magnetic separation and the obtained immune probes were stored at 4 °C for further study.

### 2.3. Preparation of the SERS-Active Immune Substrate

First, the purchased SiC sandpapers with different grain sizes (200, 240, 280, 320, 360, and 400 mesh) were used as a template to magnetron sputter Ag films as a SERS-active substrate. Afterwards, the captured antibody was immobilized on the substrate (240 mesh) by dropping 20 µL PBS solution of anti-PSA (0.2 mg/mL) and, subsequently, incubated at 4 °C for 12 h. In order to remove residual protein from the substrate, it was, then, washed with TBS/0.05% Tween 20 buffer solution, TBS, PBS, and deionized water. After that, the nonspecific adsorption points were blocked via treating the substrate with BSA at 37 °C for 2 h and washed again. The SERS-active immune substrate was finally obtained and stored at 4 °C for further experiments.

### 2.4. Recyclable Sandwich Immunoassay

**Scheme 1:**
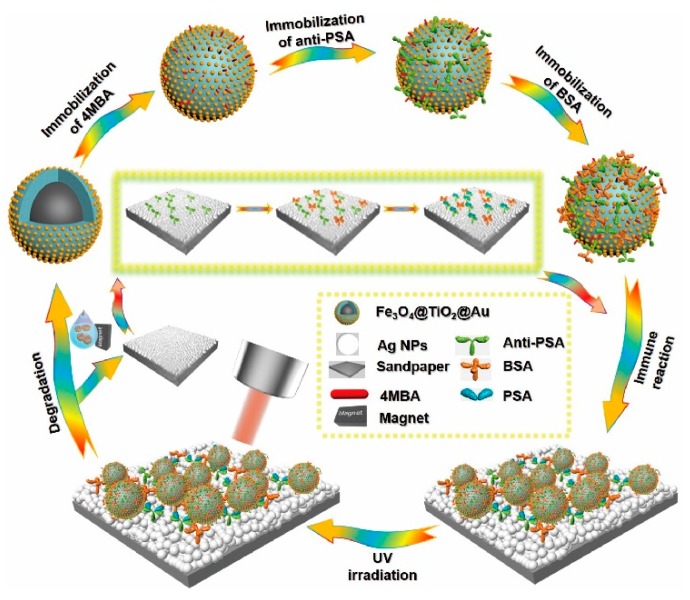
Schematic illustration of the recyclable SERS-based immunoassay.

The recyclable sandwich immunoassay was determined based primarily on the specific interaction between antigen and antibody. First, 10 µL PBS solution of PSA was dropped onto the as-prepared immune substrate before being stored at 37 °C for 2 h. Then, it was washed in TBS/0.05% Tween 20 buffer solution, TBS, PBS, and deionized water to remove the unbound antigen. After that, the immune substrate was covered with 20 µL of the Fe_3_O_4_@TiO_2_@Au immune probes and incubated at 4 °C for 2 h. Subsequently, after being washed again to remove unbound immune probes, the substrate with sandwich immune structure was dried and used for the following SERS evaluations. After the measurement, the sandwich immune structure was irradiated with an ultraviolet lamp (365 nm, 6 W/cm^2^) to degrade the antigen and antibody on the substrate. During the photocatalysis, the SERS spectra of 4MBA was monitored every 10 min until there was no characteristic signal. After the photocatalysis, in addition to the recovery of the nanocomposites after collecting under magnet, the Ag-coated sandpaper substrate was also reused after washing. Subsequently, another cycle of detection and photocatalysis of PSA was conducted by reconstructing the sandwich immunostructure composed of the previous nanocomposites and Ag-coated sandpaper substrate, as described above. These immunoassay cycles with SERS detection and photocatalysis were repeated, until a LOD of the target PSA was reached, as illustrated in Scheme 1.

### 2.5. Instruments

The morphologies of samples were investigated using a SU-70 field emission scanning electron microscopy (SEM, Hitachi, voltage: 5 kV). The energy dispersive spectroscopy (EDS) and transmission electron microscopy (TEM) images were measured on a Tecnai G2 F20 instrument (FEI). The X-ray power diffraction (XRD) data were collected with a D8 ADVANCE (Bruker). UV-Vis spectra were recorded using a TU-1901 general absorption spectrophotometer. Zeta potential distribution was performed on a Zetasizer Nano ZS90 (Malvern) with a He-Ne laser at 632.8 nm. Magnetic properties were determined with a SQUID (XL-7) at 300 K. The deposition of Ag film was performed in a TA13-XD magnetron sputtering device (pressure 6 × 10^–4^ Pa and deposition rate 0.5 Å/s). The SERS measurements were performed with a BWS415 miniature Raman spectrometer (BWTek) excited under 785 nm. Each spectrum was collected for 10 s under laser power of 20 mW. 

## 3. Results and Discussion

### 3.1. Characterization of the Fe_3_O_4_@TiO_2_@Au Immune Probe 

**Figure 1:**
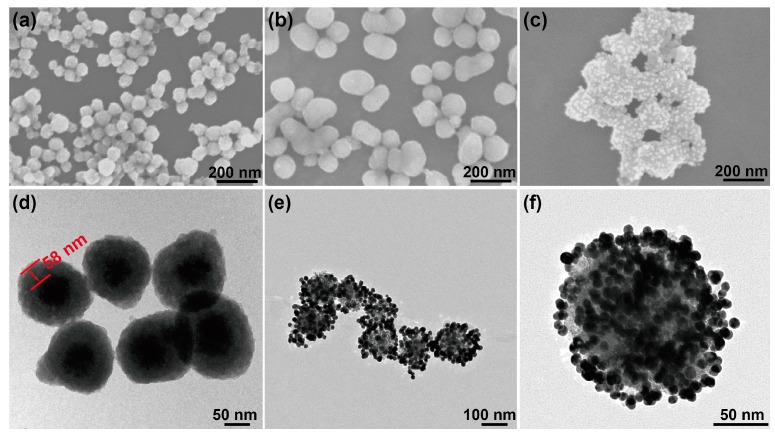
SEM images of (**a**) Fe_3_O_4_; (**b**) Fe_3_O_4_@TiO_2_; and (**c**) Fe_3_O_4_@TiO_2_@Au nanocomposites. TEM images of (**d**) Fe_3_O_4_@TiO_2_ and (**e**, **f**) Fe_3_O_4_@TiO_2_@Au nanocomposites.

**Figure 2:**
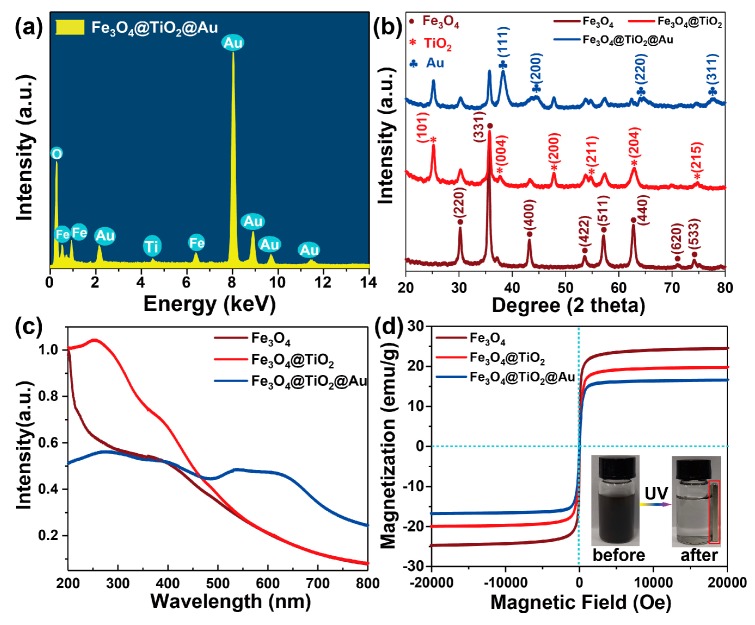
(**a**) EDS spectra of Fe_3_O_4_@TiO_2_@Au nanocomposites. (**b**) XRD patterns; (**c**) UV-vis absorption spectra; and (**d**) magnetization curves of Fe_3_O_4_; Fe_3_O_4_@TiO_2_; and Fe_3_O_4_@TiO_2_@Au nanocomposites.

The high yield of spherical Fe_3_O_4_ NPs with an average diameter of 85 ± 10 nm is presented in Figure 1a. Compared with the bare Fe_3_O_4_ core, the average diameter of the Fe_3_O_4_@TiO_2_ NPs increases to 140 ± 10 nm (Figure 1b). Afterwards, a large amount of uniform Au seeds is found to attach onto the TiO_2_ shell and lead to rough surfaces, as presented in Figure 1c. The core-shell structure of the Fe_3_O_4_@TiO_2_ NPs is clearly exhibited in Figure 1d and the thickness of the TiO_2_ is 58 nm. The average diameter of the scattered Au seeds is implied as 15 nm, as illustrated in Figure 1e,f. The EDS, as shown in Figure 2a, then reveals the chemical composition of the nanocomposites and expatiates the dominant peaks of Fe, O, Ti, and Au. In addition, XRD patterns of the Fe_3_O_4_, Fe_3_O_4_@TiO_2_, and Fe_3_O_4_@TiO_2_@Au nanocomposites are shown in Figure 2b. The specific peaks of Fe_3_O_4_ NPs can be assigned to the cubic phase of Fe_3_O_4_. After first coating, several additional peaks from anatase TiO_2_ are demonstrated. With the loading of Au seeds, the new peaks are indexed to the face-centered cubic Au phase. As it is presented in Figure 2c, the semiconductor absorption band edge of TiO_2_ appears at about 530 nm, in addition to the absorption band of Fe_3_O_4_ from 400 to 394 nm after the formation of TiO_2_ shell. With the introduction of Au seeds, their typical localized surface plasmon resonance (LSPR) band from 500 to 680 nm dominates in the absorption spectra. Moreover, the magnetic performance of Fe_3_O_4_ during the two steps of coating was investigated, as presented in Figure 2d. Although a gradual decreasing trend of the magnetization of samples was observed, enough magnetization is still kept at about 16.7 emu/g in the final Fe_3_O_4_@TiO_2_@Au nanocomposites and they could be effectively collected by the magnet, as shown in the inset of Figure 2d. In addition, as illustrated in Table 1, the surface potential of the sample first decreases from −1.4 to −22.9 mV after the formation of TiO_2_ surface instead of that of Fe_3_O_4_. Then, amino modification induces the converting of the surface from negativity to positivity, confirming by the changing of surface potential from −22.9 to 25.4 mV. Finally, the modification of negative charged Au seeds results in a dramatic decrease of the surface potential to 7.8 mV. All these characterizations identify the successful synthesis of the Fe_3_O_4_@TiO_2_@Au nanocomposites.

**Figure 3:**
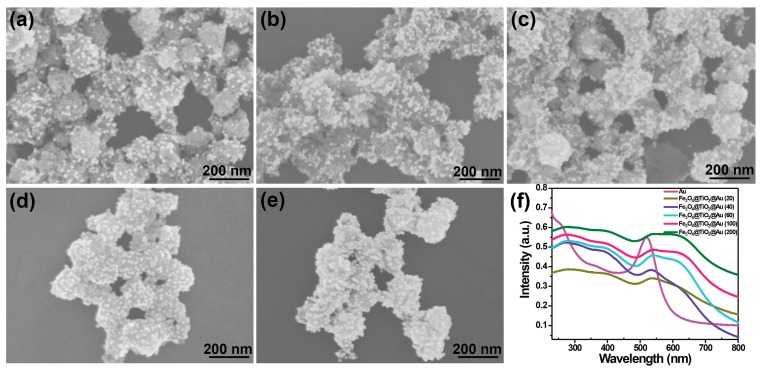
SEM images of the obtained Fe_3_O_4_@TiO_2_@Au nanocomposites synthesized under different volumes of Au seeds. (**a**) 20; (**b**) 40; (**c**) 60; (**d**) 100; and (**e**) 200 mL; and (**f**) their corresponding UV-Vis absorption spectra.

**Figure 4:**
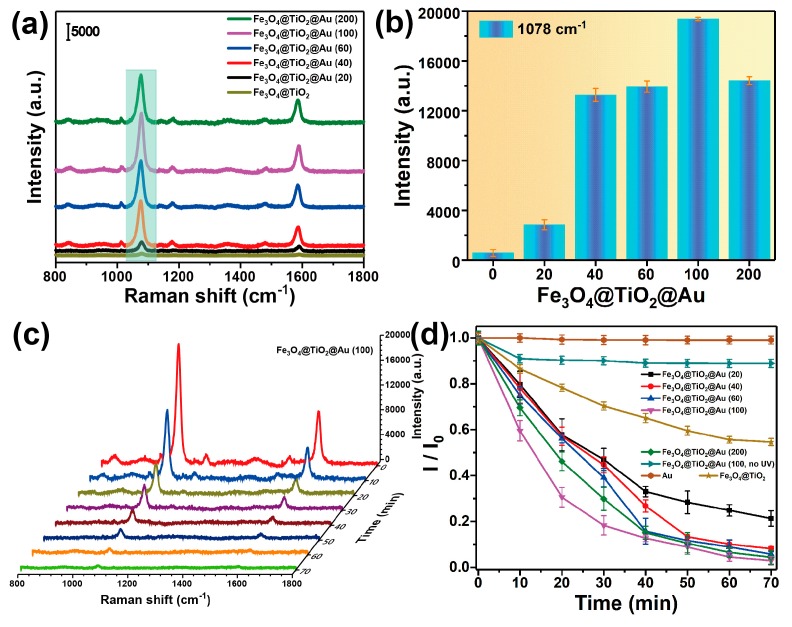
(**a**) SERS spectra of 4MBA on the Fe_3_O_4_@TiO_2_@Au nanocomposites synthesized with different volumes of Au seeds; and (**b**) columnar distribution of the SERS intensity at 1078 cm^−1^; (**c**) SERS spectral change of 4MBA on Fe_3_O_4_@TiO_2_@Au nanocomposites synthesized with 100 mL of Au seed soluiton during UV light irradiation; (**d**) Photocatalytic degradation of 4MBA (exampled by I/I_0_ of SERS peak at 1078 cm^−1^) in aqueous suspension of Au, Fe_3_O_4_@TiO_2_, and various Fe_3_O_4_@TiO_2_@Au nanocomposites with and without UV irradiation.

Owing to the rigid correlation between the optical property of Au seeds and their aggregation degree, it is necessary to fully adjust the real density of Au seeds on the Fe_3_O_4_@TiO_2_ NPs. As illustrated in Figure 3a–e, the depositing density of Au seeds gradually increased when more Au seeds were used in the electrostatic attraction process. Along with the increase of the coated Au seeds, the absorption band of the nanocomposites gradually red shifts and generates obvious broadening as identified from Figure 3f [33]. Next, the Au density-dependent SERS performance of the nanocomposites was analyzed with 4MBA as a model Raman molecule. As shown in Figure 4a,b, the intensity of the SERS spectra at 1078 cm^−1^ evidently increased with the increasing density of Au seeds, which could be attributed to the excitation wavelength approaching the typical LSPR band and the enriching of the electromagnetic “hotspots” from gradually aggregated Au seeds [34,35]. Alternatively, more surface Au seeds give rise to a more effective linkage area, which could facilitate the following adsorption of target molecules, and ensure that more charges transfer from Au to the molecules. However, after the volume of Au seed solution exceeded 100 mL, the intensity of the SERS signal was significantly weakened, which was mainly due to the excessive agglomerations of the coated Au seeds, as identified in Figure 3e, and thus leads to an obvious increase of the interparticle distance.

**Figure 5:**
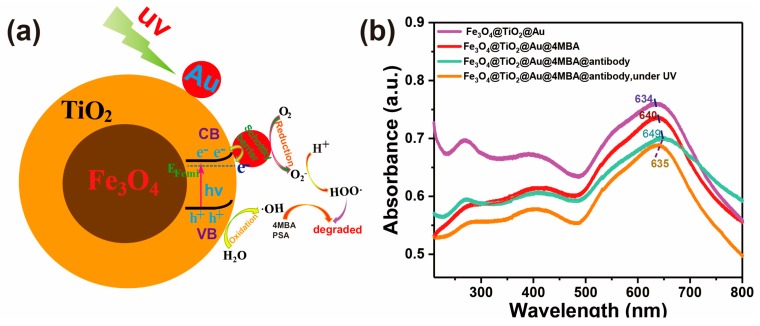
(**a**) The photocatalytic mechanism of Fe_3_O_4_@TiO_2_@Au nanocomposites; (**b**) Absorption spectra of Fe_3_O_4_@TiO_2_@Au, Fe_3_O_4_@TiO_2_@Au@4MBA, Fe_3_O_4_@TiO_2_@Au@4MBA@antibody, and Fe_3_O_4_@TiO_2_@Au recovered by UV irradiation after immunoassay.

Moreover, in addition to exploring the Au density-dependent SERS enhancement effect, their photocatalytic efficiency was also studied by degrading of 4MBA under UV irradiation in the following works. The gradual changes of the SERS spectra at various irradiation intervals are shown in Figure 4c (Figure S1a–d). With the extension of irradiation time, the SERS signal continuously slumped and only faint characteristic peaks of 4MBA could be seen after being illuminated for 60 min. Figure 4d shows the time-dependent photocatalytic degradation of 4MBA by using all these nanocomposites with different coating amounts of Au, in which I_0_ is the initial SERS intensity of 4MBA and I is that after UV irradiation. There is almost no photocatalytic degradation of 4MBA if no UV irradiation was presented, suggesting the necessity of the light source. Furthermore, the photocatalytic degradation rate of 4MBA first increased with more and more decorated Au seeds, until the sample synthesized with 100 mL of Au seed solution was used, and then it decreased conversely. The Fe_3_O_4_@TiO_2_@Au nanocomposites (100 mL of Au seed solution) exhibit the highest photocatalytic efficiency with a total degradation rate of 99.7% within 70 min UV irradiation, while the pure Fe_3_O_4_@TiO_2_ NPs show the lowest degradation rate of only about 46.6% under the identical condition. These phenomena represent the evidence for the critical role of Au seeds with rationally designed aggregation state in photocatalysis, which can be attributed to the formation of a Schottky barrier between the Au seeds and TiO_2_ (the work functions *φ*_Au_ = 5.1 eV [36] and *φ*_TiO2_ = 4.2 eV [37]). This barrier inhibits the combination of photogenerated electron holes to a certain extent, thus giving them longer time intervals to diffuse to the surface and effectively promoting the possible redox reaction, as shown in Figure 5a [22,38,39]. In addition, since the effective adsorption of 4MBA molecules is a prerequisite for the catalysis, the photocatalytic efficiency has a certain relationship with the strength of the adsorption capacity [40,41,42]. Meanwhile, the increased surface with exposed Au provides more effective areas for absorbing 4MBA molecules. When 100 mL of Au seed solution was added, the densest surface Au seeds without significant agglomerations rendered the Fe_3_O_4_@TiO_2_@Au nanocomposites enough adsorption sites for the target analytes, promoting the migration of photogenerated electron-hole pairs. However, when the amount of Au seeds continued to increase, the aggregation of the modified Au NPs induced less effective Au areas, resulting in lower photocatalytic efficiency. Thereby, the photocatalytic efficiency of nanocomposites was optimum when 100 mL of Au seed solution was added.

After that, the formation process of the immune probes was also investigated using SEM image, zeta potential measurements, and UV-Vis absorption spectroscopy. As shown in Figure S2b, after anti-PSA was linked to the Fe_3_O_4_@TiO_2_@Au nanocomposites, they showed a certain degree of agglomeration. Meanwhile, as illustrated in Table 1, the surface potential of the samples changed from 7.8 to –17.9 and to –20.2 mV, showing that the 4MBA and anti-PSA were successfully linked to the surface of Fe_3_O_4_@TiO_2_@Au nanocomposites [43]. In addition, a slight red shift of the LSPR band was observed from 634 to 640 and to 649 nm after the addition of 4MBA and anti-PSA, as shown in Figure 5b, which clearly indicated that the size of aggregates increased due to their successful attachment [31,44]. It should be noted that the aggregation of the nanocomposites after modification was not as obvious, which can be mainly ascribed to the fact that protection of TiO_2_ made the nanocomposites more stable than the bare noble metal.

### 3.2. Characterization of the SERS-Active Substrate

**Figure 6:**
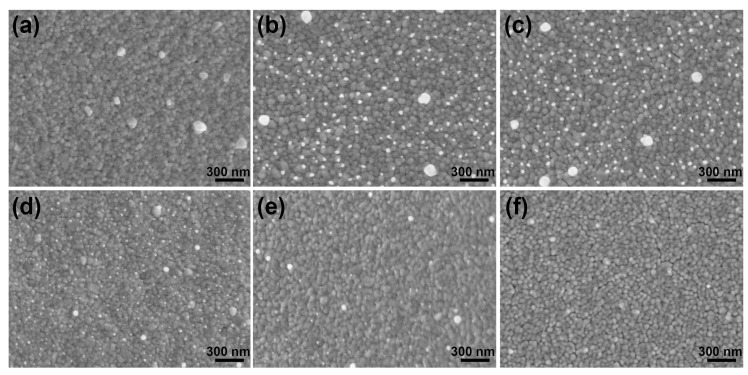
SEM images of Ag-coated sandpaper with different meshes. (**a**) 200; (**b**) 240; (**c**) 280; (**d**) 320; (**e**) 360; and (**f**) 400.

**Figure 7:**
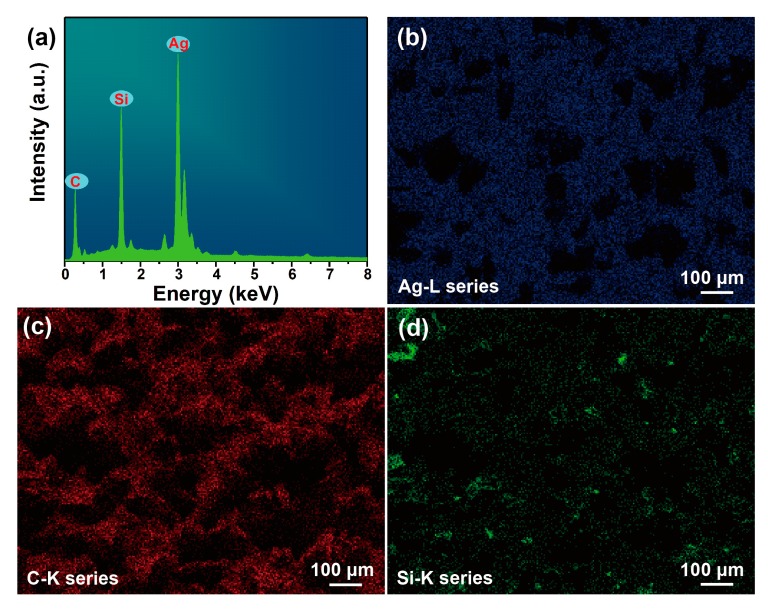
(**a**) EDS of Ag-coated sandpaper and the corresponding local elemental mapping of (**b**) Ag; (**c**) C; and (**d**) Si.

It is well known that the SERS performance of Ag film depends on its configuration parameters, for example, surface morphology, homogeneity, and roughness [45,46]. Thus, sandpapers with mesh ranging from 200 to 400 were chosen to be decorated with Ag film to serve as SERS-active substrates. Moreover, the Ag-coated sandpaper also exhibits some advantages suitable for clinical immunoassay such as low cost, good repeatability, and stability. The morphologies of the obtained Ag-coated sandpaper are characterized by SEM, as shown in Figure 6a–f. It is observed from these images that the closely packed Ag NPs homogeneously distribute on these sandpapers, giving rise to numerous ultra-narrow nanogaps. It is noted that the size of the sputtered Ag NPs first increases when the mesh of sandpaper increases from 200 to 280, and then, it reversely decreases when the mesh further increases to 400. In particular, the nanogaps gradually disappear when the Ag NPs become smaller and more significantly aggregated. Then, the dominated existence of the Ag, Si, and C elements are demonstrated by the EDS pattern (Figure 7a) and their homogeneous distributions around the whole substrate are exhibited through the elemental mapping, respectively, (Figure 7b–d).

**Figure 8:**
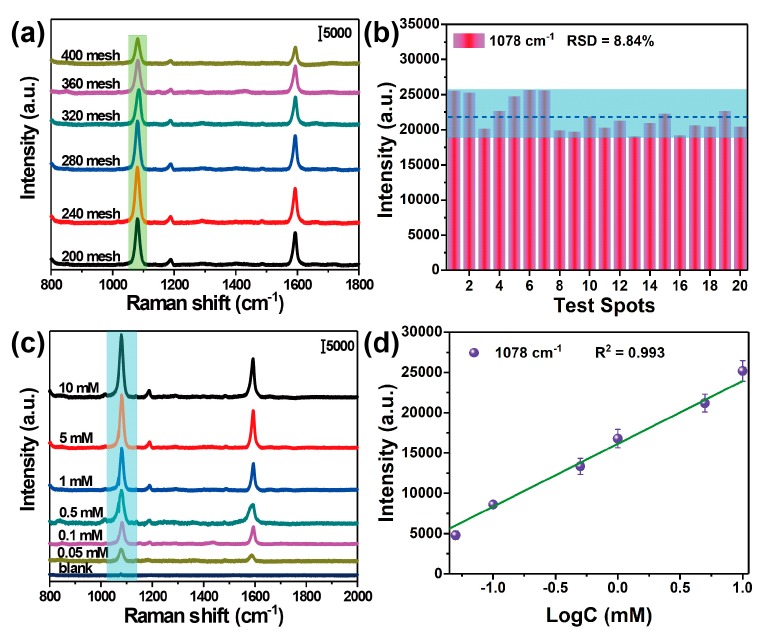
(**a**) The SERS spectra from the Ag-coated sandpaper with different mesh; (**b**) The SERS intensities at 1078 cm^−1^ from 20 random test dots on the Ag-coated sandpaper (240 mesh); (**c**) SERS spectra of 4MBA with different concentration and Raman spectra of 4MBA power; (**d**) The corresponding dose-response curve of the peak intensity at 1078 cm^−1^.

Then, the SERS spectra of 4MBA dropped onto the as-prepared substrates were measured to estimate their electromagnetic enhancement efficiency. As shown in Figure 8a, the intensity of the SERS peak at 1078 cm^−1^ increases by increasing the mesh of the sandpaper to 240, and then gradually declines. As discussed above, the larger size of the Ag NPs and the proper interparticle distance among them on the 240-mesh sandpaper induces the greatest number of “hotspots” and results in the strongest SERS signals. Therefore, the SERS-active substrate developed with the 240-mesh sandpaper acted as the optimal one and was used in the experiments that followed.

Furthermore, the enhancement factor (EF) of Ag-coated sandpaper was calculated using the following equation: *EF = (I_SERS_/I_Raman_) × (N_Raman_/N_SERS_)*, where *I_SERS_* and *I_Raman_* represent the integrated intensities of SERS peaks at 1078 cm^-1^ of 4MBA molecules attached to the Ag-coated sandpaper and its power, respectively. *N_Raman_* and *N_SERS_* represent the number of 4MBA molecules in the power and absorbed on the substrate within the laser spot, respectively. The *I_SERS_* and *I_Raman_* were calculated to be 5.34 × 10^5^ and 3.60 × 10^3^ by the SERS signal from the Ag-coated sandpaper and Raman spectrum from the power (Figure 8c). *N_Raman_* was calculated to be 2.32 × 10^11^ mainly based on the diameter (*d = (λ/NA) ×* 1.22) and the penetration depth (*h = 2λ/NA^2^*) of the focus illumination spot, where *λ* and *NA* are 785 nm and 0.4, respectively. *N_SERS_* was determined by the equation *N_SERS_ = N_A_ × A × δ*, where *N_A_* is the Avogadro constant, *A* is the effective area occupied by the monolayer 4MBA molecules within the laser spot of 4.52 µm^2^ [47], and *δ* is estimated to be 2.0 × 10^9^ cm^2^/mol according to the molar amount of 4MBA molecules per square centimeter [48,49]. Thereby, *N_SERS_* was 1.35 × 10^7^. Ultimately, Ag-coated sandpaper yields the EF value which was calculated to be 2.55 × 10^6^.

In order to evaluate the homogeneity of the SERS signal from the as-obtained Ag-coated sandpaper, intensities of the SERS peak of 1078 cm^−1^ from 20 randomly selected points on the substrate are illustrated, as shown in Figure 8b. The calculated relatively standard deviation (RSD) is 8.84%, indicating the excellent uniformity of the optimal substrate. In addition, the solutions of 4MBA with concentration from 10 to 0.05 mM were measured, and the intensity of 1078 cm^−1^ peak obviously drops with the dilution of 4MBA, as shown in Figure 8c. It should be pointed out that each spectrum was averaged from ten randomly selected data. Moreover, Figure 8d displays the linear change of SERS intensity as a function of 4MBA concentration, and the LOD was calculated to be 0.014 mM in accordance to the three time of signal-to-noise ratio from the blank substrate [50,51].

### 3.3. Recyclable SERS-Based Immunoassay of PSA

**Figure 9:**
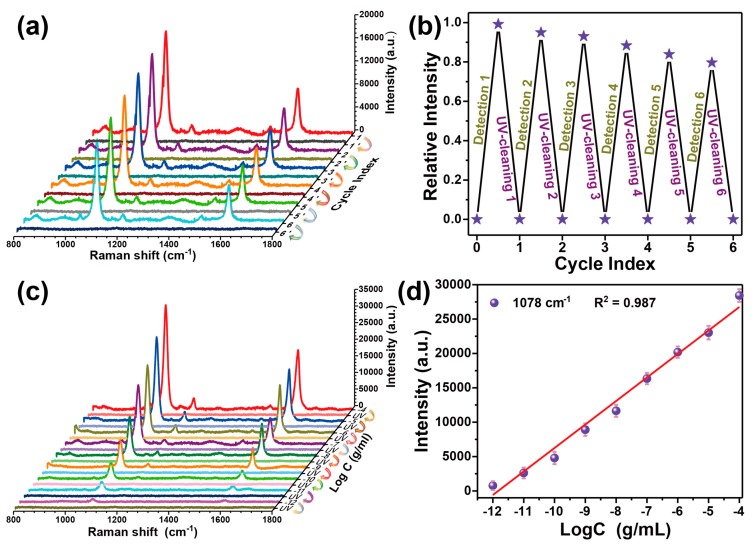
(**a**) SERS spectra recorded during recyclable immune detection of PSA at the concentration of 10^−7^ g/mL; (**b**) The corresponding variation of the relative SERS intensity at 1078 cm^−1^; (**c**) Recycle immunoassays of PSA at different concentrations based on SERS spectra; (**d**) The corresponding variation of the SERS intensity at 1078 cm^−1^.

In previous literatures, the SERS-based immunoassay of PSA has been widely reported. However, there are almost no systematical investigations of recyclable SERS-based immunoassay with the introduction of photocatalytic materials. Herein, through constructing sandwich immune structures with Fe_3_O_4_@TiO_2_@Au nanocomposites and Ag-coated sandpaper, we made a thorough inquiry on the prospect of the recyclable SERS-based immunoassay. The assay and photocatalysis of PSA with the concentration of 10^-7^ g/mL were chosen as a model to clarify if the catalytic cleaning of target antigen and modified antibody after immunoassay could be achieved, and how many effective cycles could be achieved. First, the immune probes based on the Fe_3_O_4_@TiO_2_@Au nanocomposites were successfully captured on the immune substrate using the Ag-coated sandpaper, as illustrated in Figure S3a,b. After UV irradiation and washing, the disappearance of probes from the substrate indicate that the antigen and antibody have been degraded during photocatalysis (Figure S3c). Meanwhile, the hypothesis is also supported by the eliminating of the characteristic SERS signal of 4MBA from the substrate, as shown in Figure 9a,b, confirming the feasibility of recyclable immunoassay. Moreover, the SEM image of recovered Fe_3_O_4_@TiO_2_@Au nanocomposites utilizing the magnetic properties of Fe_3_O_4_ (Figure S3d) shows no significant difference to that before immunoassay, indicating that it has good stability, and their absorption spectra present an obvious blue shift from 649 to 635 nm (Figure 5b) together with a change of the surface potential from −20.2 to 4.8 mV (Table 1). All these phenomena suggest the complete degradation of the 4MBA and anti-PSA from the surface of Fe_3_O_4_@TiO_2_@Au nanocomposites.

Additionally, to probe into the cycling efficiency and stability of nanocomposites in immunoassay, the detection and photocatalytic eliminating of PSA were carried out six times, as illustrated in Figure 9a. The appearance of strong SERS signals after the immunoassays (1 to 6), and their removing after UV illumination (1’ to 6’), were successfully proven. The drift in the SERS intensity by 20% after 6 cycles of immunoassay and photocatalytic reaction can be visualized in Figure 9b, which verifies the limited cycle times for maintaining enough enhancement ability. The performance of the immune structure in the recyclable detection of PSA with diluted concentration was further investigated. As shown in Figure 9c, the intensity of SERS spectra decreases by degrees with the reduction of PSA. Then, a standard curve for the recyclable detection of PSA is plotted in Figure 9d, which is primarily based on the intensity of 4MBA at 1078 cm^−1^ against the logarithm of PSA concentration over the range of 10^−4^ to 10^−12^ g/mL, showing an admirable correlation (R^2^ = 0.987). The error bar is the standard deviation calculated from measuring ten sets of data. Finally, the LOD was determined to be 1.871 pg/mL.

**Figure 10:**
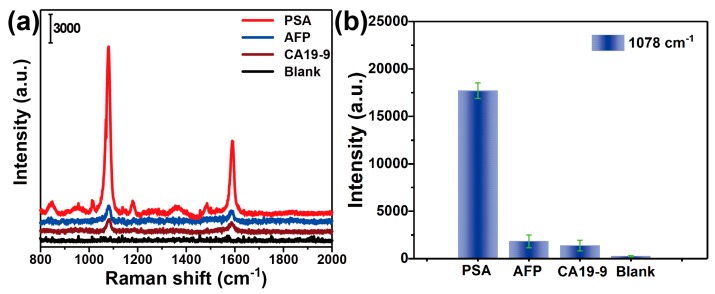
(**a**) SERS spectra of 4MBA in the specific immunoassay for PSA, AFP, and CA19-9, and the background signals without any target antigen; (**b**) Columnar distribution of the SERS peak intensity at 1078 cm^−1^.

In addition, the nonspecific antigen AFP and CA19-9 were also tested with the above immune probe and substrate, and the results are shown in Figure 10a,b. Although there are still some faint SERS signals for AFP and CA19-9 induced by nonspecific physical adsorption different from the background noises, it is nearly negligible as compared with the signal from detecting PSA with the same concentration. This proves that the SERS-based immunoassay has high sensitivity and specificity for target antigens. Consequently, the developed recyclable SERS-based immunoassay provided a new strategy for rapid and cost-effective detection of cancer biomarker, which could have potential to be applied in subsequent clinical diagnosis. This novel recyclable immunoassay is expected to be applied for the detection of tumor makers in the clinical serum sample of cancer patients.

## 4. Conclusions

In summary, the recyclable detection of PSA was successfully achieved based on only one batch of the sandwich immune structure, which is combined with multifunctional Fe_3_O_4_@TiO_2_@Au nanocomposites and Ag-coated sandpaper. In the first place, the structure-dependent SERS and photocatalytic performance of nanocomposites was systematically investigated by adjusting the density of outside Au seeds on Fe_3_O_4_@TiO_2_ NPs, and the optimum performance was found in the samples prepared by employing 100 mL Au seed solution. Then, the SERS enhancement capability of Ag-coated sandpaper with different meshes was evaluated by monitoring the SERS spectra of 4MBA, and the LOD of 0.014 mM was achieved. Afterwards, the feasibility of the recyclable immunoassay was approved by the variation of zeta potential measurements, absorption spectra, and SEM images before and after photocatalysis. And especially, the drift in the SERS intensity could still be limited within 20% after six cycles of detection and photocatalysis. Thus, the developed sandwich immune structure was applied to recyclably analyze PSA with different concentrations in the range of 10^−4^ to 10^−12^ g/mL, and the LOD was calculated to be as low as 1.871 pg/mL. In addition, the nonspecific adsorption measurements for the monitoring of AFP, PSA, CA19-9 antigens confirmed the specificity of the obtained detection structure. In summary, this recyclable strategy has promising prospects in applications of clinical measurements for the diagnosis of cancer.

## Figures and Tables

**Table 1:** Zeta potential measurements.

SERS Probes	Zeta Potential/mV
Fe_3_O_4_	−1.4 ± 0.1
Fe_3_O_4_@TiO_2_	−22.9 ± 0.4
Fe_3_O_4_@TiO_2_@NH_2_	25.4 ± 0.5
Fe_3_O_4_@TiO_2_@Au	7.8 ± 0.8
Fe_3_O_4_@TiO_2_@Au@4MBA	−17.9 ± 1.2
Fe_3_O_4_@TiO_2_@Au@4MBA@anti-PSA	−20.2 ± 1.1
Fe_3_O_4_@TiO_2_@Au@4MBA@anti-PSA, under UV	4.8 ± 0.1

## Supplementary Materials

The following are available online at https://www.mdpi.com/2079-6374/10/3/25/s1, Figure S1: (a) SERS spectral change of 4MBA on the Fe_3_O_4_@TiO_2_@Au NPs synthesized with (a) 20; (b) 40; (c) 60; and (d) 200 mL Au NPs during UV light irradiation, Figure S2: SEM images of (a) blank sandpaper and (b) Fe_3_O_4_@TiO_2_@Au with the modification of anti-PSA, Figure S3: (a and b) SEM images under different magnification of the prepared sandwich immune structure with 10^−7^ g/mL PSA; (c and d) SEM image of the immune substrate and Fe_3_O_4_@TiO_2_@Au (100) immune probe after UV light irradiation.
