# Supplementary material for: Recyclable SERS-Based Immunoassay Guided by Photocatalytic Performance of Fe3O4@TiO2@Au Nanocomposites
Source: Biosensors (Basel). 2020 Mar 16;10(3):25. doi: 10.3390/bios10030025 (PMC7146594; doi:10.3390/bios10030025)
Supplement: Supplementary file 1 [file biosensors-10-00025-s001.pdf]

Supplementary data

Recyclable label-free SERS-based immunoassay of PSA in human serum mediated by enhanced photocatalysis arising from Ag nanoparticles and external magnetic field

Yuanyuan Du[a], *Hongmei Liu*[b], *Ying Chen*[a], *Yiran Tian*[a], *Xiaoling Zhang*[c], *Chenjie Gu*[a], *Tao Jiang*[a,*], *Jun Zhou*[a]

[a] Department of Microelectronic Science and Engineering, School of Physical Science and Technology, Ningbo University, Ningbo 315211, Zhejiang, P. R. China

[b] Institute of Solid State Physics, Shanxi Datong University, Datong 037009, Shanxi, P. R. China

[c] Department of Neurology, Ningbo Medical Treatment Centre Lihuili Hospital, Ningbo 315000, Zhejiang, P. R. China

E-mail: jiangtao@nbu.edu.cn (T Jiang)

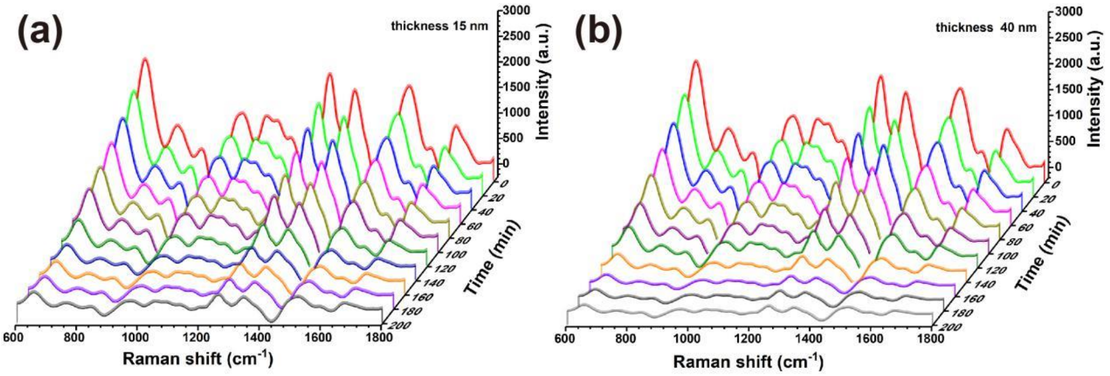

Fig. S1. SERS spectral change of PSA on Fe_3O_4@TiO_2@Ag MPs with (a) 15 and (b) 40 nm TiO_2 shell during UV light irradiation.

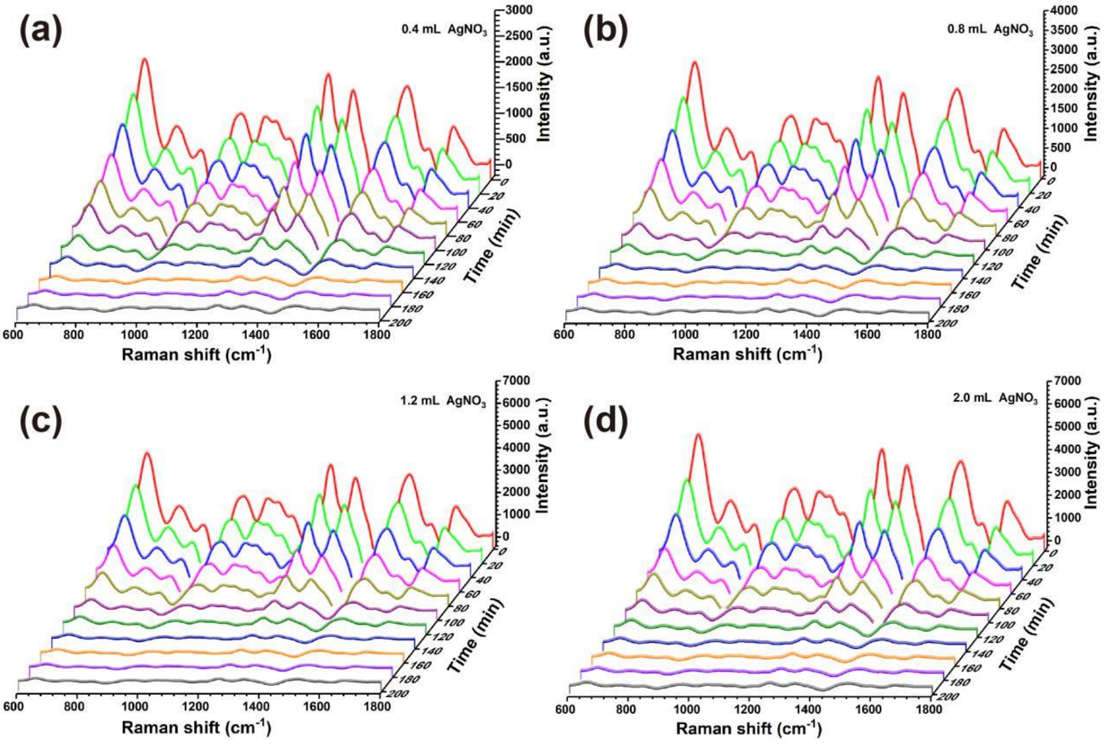

Fig. S2. SERS spectral change of PSA on Fe_3O_4@TiO_2@Ag MPs synthesized with (a) 0.4, (b) 0.8, (c) 1.2, and (d) 2.0 mL of $AgNO_3$ during UV light irradiation without magnetic field.

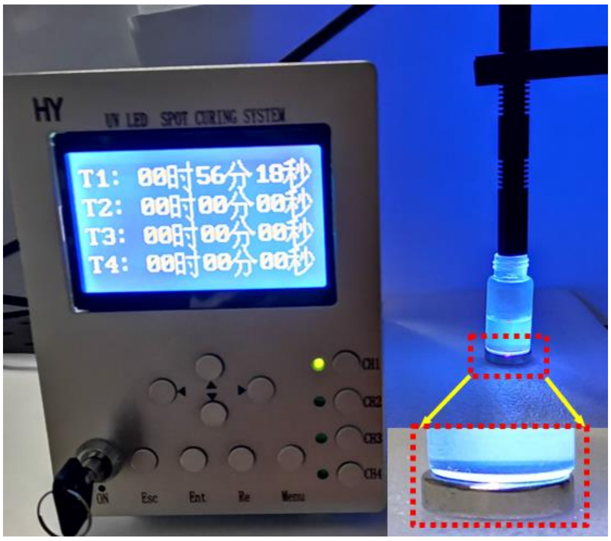

Fig. S3. A photogram of photocatalysis reaction.
